# Case Report: Elevated Lp(a) as a cause of severe ASCVD in a healthy veteran athlete with low calculated QRISK3 score

**DOI:** 10.3389/fcvm.2025.1688597

**Published:** 2026-01-13

**Authors:** Jonathan Clark-McKellar, Tracey Keteepe-Arachi, Peter Fitzgerald, Mark W. Ruddock

**Affiliations:** 1 Andarta Health and Performance, London, United Kingdom; 2 Randox Laboratories Ltd., Crumlin, United Kingdom

**Keywords:** atherosclerotic cardiovascular disease, case report, lipoprotein(a), QRISK3, screening

## Abstract

Elevated plasma lipoprotein(a) [Lp(a)] is a causal risk factor for the development of atherosclerotic cardiovascular disease (ASCVD). However, commonly used ASCVD clinical risk-assessment tools in primary care do not include the measurement of Lp(a) levels, potentially under-estimating individual risk. Here we describe the case of a late-40s, asymptomatic, normotensive, non-smoking veteran athlete with a moderately raised low density lipoprotein cholesterol (LDL-C) level and a calculated 10-year QRISK3 score of 4.1%. Despite his low calculated QRISK3 score, significantly elevated Lp(a) levels led to advanced cardiovascular imaging, which revealed severe stenosis (75%, CAD-RADS 4A) of the left anterior descending coronary artery. This case demonstrates the limitations of conventional cardiovascular risk tools and highlights the importance of Lp(a) measurement for identifying and managing high-risk patients.

## 1 Introduction

Despite improvements in lipid management and other therapeutic advances, atherosclerotic cardiovascular disease (ASCVD) remains the leading cause of global mortality. Therapeutic lowering of low-density lipoprotein cholesterol (LDL-C) levels significantly reduces ASCVD risk (1). However, residual risk remains in some patients, even when LDL-C and other traditional risk factors such as hypertension are adequately controlled (2). Consequently, there is a need to identify and manage additional contributors to residual ASCVD risk not captured by current clinical risk calculators (2–4).

Lipoprotein(a) [Lp(a)] is a low-density lipoprotein (LDL)-like particle synthesized in the liver, consisting of LDL covalently bonded to a unique glycoprotein, apolipoprotein(a) [Apo(a)], which forms characteristic ‘Kringle' domains around the LDL particle. Elevated plasma Lp(a) levels have been identified as a causal risk factor for ASCVD (5, 6). Lp(a) exerts pro-thrombotic, pro-inflammatory, and pro-atherogenic effects (7), which are associated with calcific aortic stenosis and peripheral vascular disease (8, 9). Notably, ASCVD risk associated with elevated Lp(a) persists independently of plasma levels of other Apolipoprotein B (ApoB)-100-containing LDL particles, whether influenced by genetic factors or therapeutic interventions (10).

Plasma Lp(a) concentrations are predominantly genetically determined by variations in the LPA gene, which influence Apo(a) isoform size and particle number (11). A single lifetime measurement of Lp(a) is usually sufficient to identify individuals at risk, however, various non-genetic factors can influence Lp(a) concentrations, including co-morbidities such as hypothyroidism, chronic kidney disease, and medications such as protease inhibitors (12).

Establishing a definitive threshold for elevated Lp(a) associated with increased ASCVD risk has been challenging. Isoform size variation, particularly involving polymorphisms in the Kringle IV type-2 domain, affects the relationship between measured mass and particle number, complicating standardisation efforts (11). Historically, measurement in mg/dL has proven imprecise for clinical thresholds, and contemporary guidelines advocate measurement in nmol/L to more accurately reflect particle number and clinical risk (12).

Currently, North American and European clinical guidelines suggest adopting thresholds ranging from 30 to 50 mg/dL, or 75, 100, or 125 nmol/L (12–14). Using a threshold of 75 nmol/L, approximately 25% of Caucasians, 50% of individuals of West African descent, and 10% of Japanese individuals would be classified as having increased cardiovascular risk (15).

Despite increasing recognition of elevated Lp(a) as a significant risk factor, UK National Institute for Health and Care Excellence (NICE) guidelines currently do not recommend routine Lp(a) measurement in standard ASCVD risk assessment (16).

## 2 Case presentation

An asymptomatic Caucasian male in his late 40s presented for pre-exercise participation cardiac screening, ahead of planned participation in several ultra-endurance events, including an extreme high-altitude mountain summit within the next 18 months.

The patient was non-diabetic, normotensive, and a lifelong non-smoker, with no significant personal cardiac history. The patient had no known family history of ASCVD or no known genetic disorders that may influence their risk. As a former professional boxer with extensive experience in ultra-endurance events, he maintained excellent cardiovascular fitness.

Physical examination revealed a muscular build with elevated body mass index (BMI) at 33.46 (height 172 cm, weight 99 kg), though no other features of metabolic syndrome; a waist circumference of 96.5 cm, office blood pressure of 118/72 mmHg, and normal glycated haemoglobin (HbA1c), triglycerides, and fasting insulin levels (Supplementary Table S1). An electrocardiogram (ECG) showed sinus bradycardia at 52 bpm without evidence of ischemia.

### 2.1 Investigations

**Figure 1:**
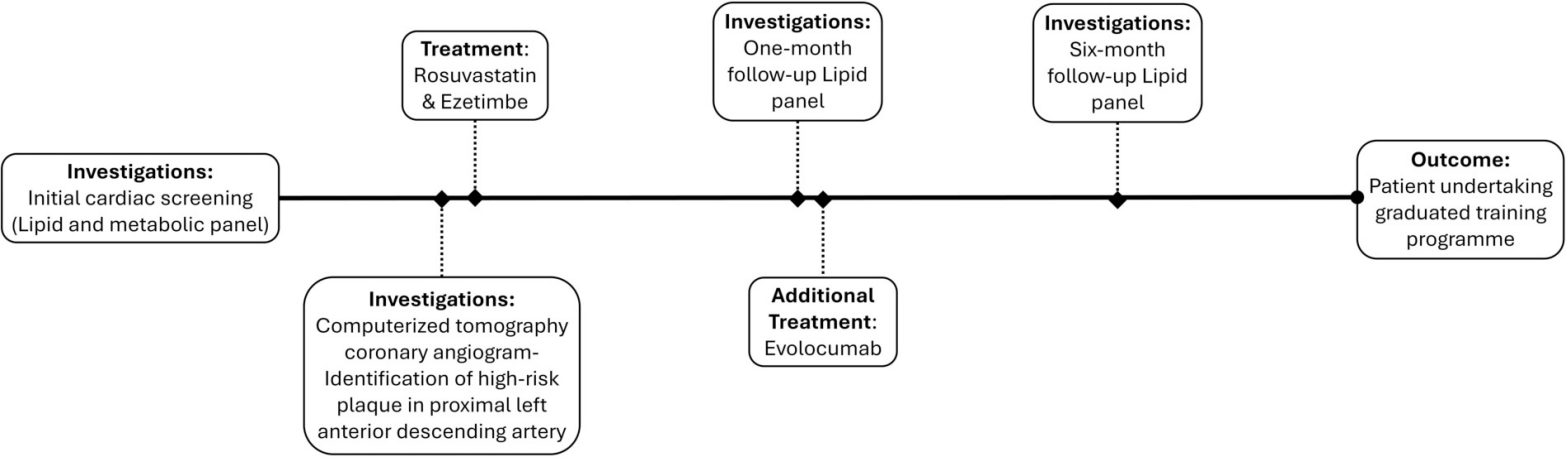
Overview of patient pathway.

**Figure 2:**
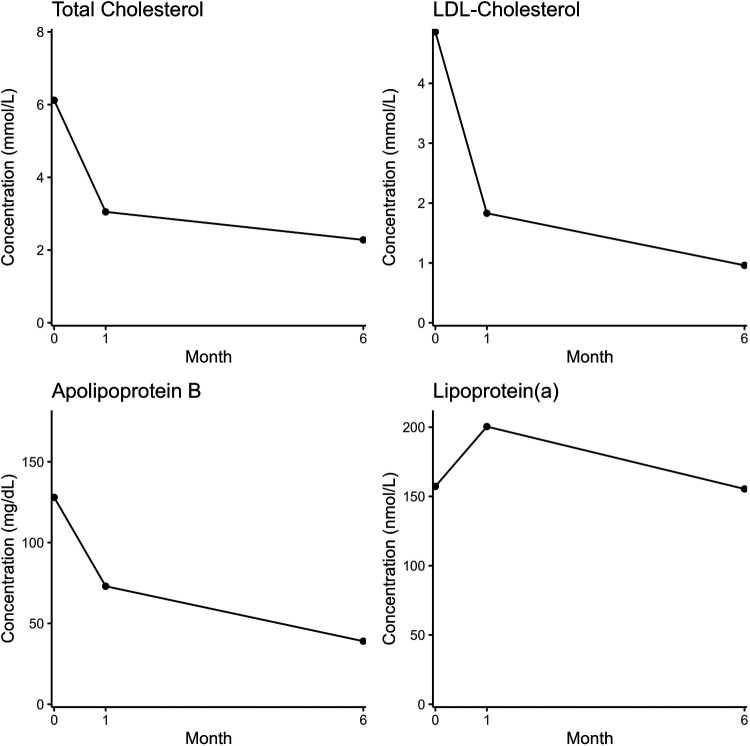
Biomarker concentrations at initial patient presentation, 1 month following treatment and 6 months follow-up.

As part of the patient's cardiac screening profile, a full lipid and metabolic blood profile were ordered (Figure 1). The patient's results indicated an elevated total cholesterol, LDL cholesterol, lipoprotein(a) and a low HDL-cholesterol (Figure 2). All other biomarkers (triglycerides, apoB, HbA1c, fasting insulin and random glucose) were within the normal reference range.

The patient's 10-year QRISK3 score (https://qrisk.org/index.php) was calculated at 4.1%, indicating a low 10-year risk of cardiovascular events. This compared with a risk of 3.2% for a healthy person of the same age, sex and ethnicity i.e., a BMI within normal range and TC/HDLC ratio of 4.0.

QRISK3-Lifetime Assessment (https://www.qrisk.org/lifetime/) indicated a 49% lifetime risk of cardiovascular disease by age 99. The projected risk decreased to 39.4% if all identified modifiable risk factors (elevated BMI and cholesterol ratio) were optimally managed, with a notable increase in risk commencing only after the age of 63.

**Figure 3:**
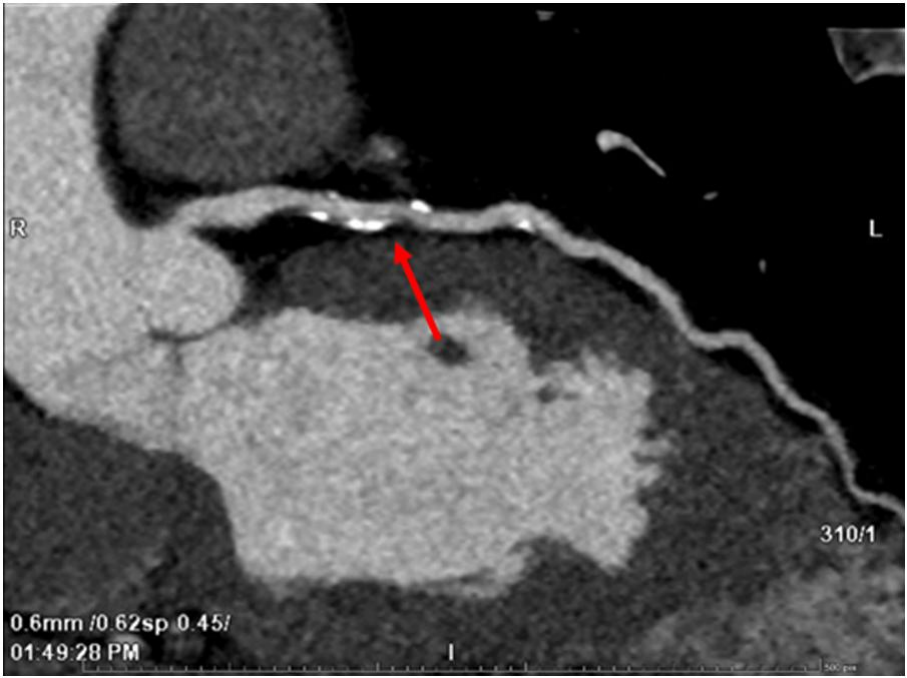
Focal mixed calcified and low attenuation plaque (red arrow) causing 75% stenosis in mid left anterior descending (LAD) artery.

Despite a low calculated cardiovascular risk, significantly elevated Lp(a) prompted further investigation. Following patient consultation, a computerized tomography coronary angiogram (CTCA) was performed, revealing a short mixed low attenuation and calcified high-risk plaque causing 75% stenosis in the proximal left anterior descending (LAD) artery (CADS-RADS 4) (Figure 3) (15). Additionally, multiple soft and calcified plaques causing up to 25% stenosis were observed in the left main and left circumflex coronary arteries. No calcification was identified on the aortic valve.

### 2.2 Treatment

The patient was commenced on lipid-lowering therapy for secondary prevention of ASCVD. Following a detailed discussion regarding patient preferences, potential side effects, and prognosis, treatment with Rosuvastatin 20 mg daily and Ezetimibe 10 mg daily was initiated. After one month, a follow-up lipid panel revealed improved total cholesterol and LDL-cholesterol (Figure 2). The level of HDL-cholesterol remained low and Lp(a) remained elevated. All other biomarkers were within the reference range (Figure 2).

Given the patient's significant coronary artery disease and persistently elevated Lp(a), additional lipid-lowering therapies were discussed. The patient opted to initiate Evolocumab 140 mg administered bi-weekly.

### 2.3 Outcome and follow-up

At six months, repeat lipid profiling demonstrated total cholesterol and triglycerides had remained normal, HDL-cholesterol remained low, Lp(a) had returned to the original level and apoB and LDL-cholesterol were now below the reference range (Figure 2).

He has remained asymptomatic and has undertaken further functional assessments, including a cardiopulmonary exercise test, which showed a VO_2_ max of 30 ml/min/kg without evidence of ischaemia. He has now begun a graduated training programme to return to full fitness, though has been strongly advised against strenuous activity in a hypoxic environment.

## 3 Discussion

Renkens et al. (17) recently presented a case series involving four patients with varying severities of ASCVD, ranging from angina to cardiac arrest, all of whom had elevated Lp(a) levels. Importantly, each of these patients also possessed significant traditional cardiovascular risk factors such as smoking, hypertension, or hypercholesterolemia requiring treatment. This contrasts with our reported case, which demonstrates significant ASCVD in an otherwise healthy, non-smoking, normotensive former athlete without other major risk factors. To our knowledge, no similar reports highlight severe ASCVD driven predominantly by elevated Lp(a), detected in primary care, in a comparable low-risk patient demographic. This case therefore highlights potential gaps in current UK ASCVD risk assessment guidelines, specifically regarding the omission of routine Lp(a) measurement for accurate cardiovascular risk assessment and tailored intervention planning.

We believe that elevated Lp(a) was a causal factor in this patient's condition, although his moderately raised LDL-C level of 4.86 mmol/L (ApoB 128 mg/dL) could have contributed. Nevertheless, lipid-lowering therapy would likely not have been initiated based solely on conventional risk calculators (18). However, detection of elevated Lp(a) prompted advanced imaging, revealing a high-risk coronary lesion—greater than 70% stenosis with low-attenuation plaque—indicating significantly increased short- to mid-term risk of acute coronary syndrome (19, 20). This finding led to aggressive lipid-lowering therapy and medical advice against undertaking high-altitude mountain climbing. Without this, it is plausible that this patient could have experienced a cardiovascular event during high-intensity exercise in a hypoxic environment or during routine activity within the next six years, contrary to the low risk suggested by his QRISK3 score.

Elevated Lp(a) levels have been implicated in the progression of low-attenuation plaques, a hallmark of high-risk coronary lesions (21, 22). Lp(a) and its associated oxidized phospholipids (OxPL) induce inflammation, cellular apoptosis, and necrosis, thus promoting the formation and progression of necrotic core (low-attenuation) plaques (7). Post-mortem studies further support the association between elevated Apo(a) and OxPL levels and advanced coronary plaque characteristics (23). Consequently, the radiological findings in this case strongly implicate elevated Lp(a) as a critical factor in early plaque development.

Measurement of Lp(a) at least once in adults is now recommended by the European Atherosclerosis Society and other international lipid societies, particularly in patients with a family history of premature cardiovascular disease or unexplained ASCVD (12). Although specific thresholds for therapeutic action remain under review, consensus is forming that markedly elevated Lp(a) levels warrant a more aggressive treatment approach. However, the decision to initiate therapy can be challenging. CTCA may play a critical adjunctive role in such cases, offering anatomical risk stratification to guide clinical decision-making. That said, the broader economic implications of routine CTCA or other imaging must be thoughtfully evaluated.

Statin therapy has been observed to modestly elevate Lp(a) in some patients, as occurred here with Rosuvastatin (24). While the mechanism remains unclear, possibly involving altered hepatic clearance, the clinical impact of this paradox requires further investigation. Importantly, statins significantly reduce cardiovascular risk through substantial LDL-C and ApoB lowering, which likely offsets any minor rise in Lp(a) (25).

At present, no licensed therapies specifically target Lp(a), which may underlie reluctance to incorporate it into widespread screening protocols. However, PCSK9 inhibitors have demonstrated modest Lp(a)-lowering effects and substantial benefits in patients with established ASCVD (3). In this patient, Evolocumab successfully restored Lp(a) to baseline, illustrating its value as part of a combination lipid-lowering strategy. Recently Lepodisiran—a small interfering RNA that suppresses hepatic Lp(a) synthesis –caused impressive reductions in Lp(a) levels in a phase 2 trial (ALPACA) and may offer a future therapeutic avenue, pending outcome data from phase 3 trials (26).

This case supports the inclusion of routine Lp(a) measurement in primary care to identify patients at increased risk of ASCVD who may benefit from individualised, aggressive lipid-lowering strategies. It highlights the necessity of refining traditional cardiovascular risk stratification models to incorporate biochemical markers such as Lp(a), facilitating more precise and effective prevention and management of cardiovascular disease. As a case report, this study is limited in its generalisability. Incorporation of routine Lp(a) into standard cardiovascular risk assessments is likely to highlight similar cases where risk would be underestimated without consideration of Lp(a). Additionally, although CTCA was used in this study to identify stenosis, many patients would not receive CTCA, based on current UK guidelines.

## 4 Patient perspective

“Knowing about this has changed everything. I had always thought of myself as healthy and believed that I was taking good care of myself. But, learning that I have heart disease and am at increased risk has made me rethink everything—from sleep, to diet to how I train, to the medication that I take. It's made me realise how short life can be if we’re not careful. I'd encourage everyone, no matter how fit and healthy you think you might be to get checked”.

## Data Availability

The raw data supporting the conclusions of this article will be made available by the authors, without undue reservation.
