# Supplementary material for: Case Report: Elevated Lp(a) as a cause of severe ASCVD in a healthy veteran athlete with low calculated QRISK3 score
Source: Front Cardiovasc Med. 2026 Jan 13;12:1688597. doi: 10.3389/fcvm.2025.1688597 (PMC12835392; doi:10.3389/fcvm.2025.1688597)
Supplement: Supplementary file 1 [file Table1.docx]

|  | Initial screen | One month post treatment | Six-month follow-up |
| --- | --- | --- | --- |
| Total Cholesterol | 6.12 mmol/L | 3.05 mmol/L | 2.28 mmol/L |
| LDL Cholesterol | 4.86 mmol/L | 1.83 mmol/L | 0.96 mmol/L |
| HDL Cholesterol | 1.09 mmol/L | 0.96 mmol/L | 1.06 mmol/L |
| Lipoprotein(a) | 157.2 nmol/L | 200.4 nmol/L | 155.4 nmol/L |
| Triglycerides | 1.42 mmol/L | 0.69 mmol/L | 1.17 mmol/L |
| Apolipoprotein B | 128 mg/dL | 73 mg/dL | 39 mg/dL |
| HbA1c | 35.8 mmol/L | - | - |
| Fasting insulin | 40.3 pmol/L | - | - |
| Fasting glucose | 4.18 mmol/L | - | - |

Supplementary Table 1: Biomarker results from the initial health screen, one month after starting treatment, and a six month follow up appointment.
